# Development of Gas Sensor Array for Methane Reforming Process Monitoring

**DOI:** 10.3390/s21154983

**Published:** 2021-07-22

**Authors:** Dominik Dobrzyniewski, Bartosz Szulczyński, Tomasz Dymerski, Jacek Gębicki

**Affiliations:** 1 Department of Process Engineering and Chemical Technology, Faculty of Chemistry, Gdańsk University of Technology, 11/12 G, Narutowicza Str., 80-233 Gdańsk, Poland; domdobrz@student.pg.edu.pl (D.D.); bartosz.szulczynski@pg.edu.pl (B.S.); jacgebic@pg.edu.pl (J.G.); 2 Department of Analytical Chemistry, Faculty of Chemistry, Gdańsk University of Technology, 11/12 G, Narutowicza Str., 80-233 Gdańsk, Poland

**Keywords:** gas sensors, sensor array, principal component regression, methane reforming, process monitoring

## Abstract

The article presents a new method of monitoring and assessing the course of the dry methane reforming process with the use of a gas sensor array. Nine commercially available TGS chemical gas sensors were used to construct the array (seven metal oxide sensors and two electrochemical ones). Principal Component Regression (PCR) was used as a calibration method. The developed PCR models were used to determine the quantitative parameters of the methane reforming process: Inlet Molar Ratio (IMR) in the range 0.6–1.5, Outlet Molar Ratio (OMR) in the range 0.6–1.0, and Methane Conversion Level (MCL) in the range 80–95%. The tests were performed on model gas mixtures. The mean error in determining the IMR is 0.096 for the range of molar ratios 0.6–1.5. However, in the case of the process range (0.9–1.1), this error is 0.065, which is about 6.5% of the measured value. For the OMR, an average error of 0.008 was obtained (which gives about 0.8% of the measured value), while for the MCL, the average error was 0.8%. Obtained results are very promising. They show that the use of an array of non-selective chemical sensors together with an appropriately selected mathematical model can be used in the monitoring of commonly used industrial processes.

## 1. Introduction

The effects of carrying out all kinds of industrial processes are not always characterized by favorable influence on the environment. Increasingly stringent restrictions require industrial plants to constantly improve the analysis and process monitoring techniques. For this purpose, measurement techniques enabling quantitative and qualitative analysis are used, which focused mainly on the use of modern analytical techniques such as gas chromatography. The application of this type of method in an industrial plant carries the consequences of high investment costs and the necessity to provide appropriate infrastructure for the proper operation of the analyzers. In the case of gas samples, it is becoming more and more popular to design analyzers based on non-selective gas sensor arrays [1,2]. Matrices constructed in this way provide, among other things, a holistic analysis of the composition of the gas mixtures without separating them into individual components and shortening their duration. The most important advantage of this type of solution is the low price, which goes hand in hand with the simplicity of use. The latest applications of gas sensor arrays for the control and monitoring of industrial processes are presented in Table 1.

It should be objectively stated that all available analytical (instrumental) techniques will have smaller or larger defects affecting the quality of the analysis performed with their application. It is assumed that in order to develop an innovative and effective industrial processes monitoring system, a complementary and integrated approach, based on the synergy of their operation, is necessary [17,18]. Undoubtedly, this will ensure a full and comprehensive assessment of a given process sample, taking into account all possible factors, but on the other hand, many of these techniques have advantages that allow them to be used independently for specific tasks [19,20]. Table 2 shows the strengths and weaknesses of the use of sensor arrays for the analysis of process gas samples compared to the gas chromatography method.

Gas chromatography is widely used in the measurement, management, monitoring and control of processes carried out in breeding swine [21], municipal wastewater plants [22], municipal landfills [23] or even in the food industry, for example, in the production of cognac [24].

Recent research has been carried out intensively in the field of gas sensor arrays, as it has been observed that they can be used for continuous monitoring of industrial processes [25]. Due to the easily replaceable modules, it is possible to adapt the matrix to the current technological process, and the lack of selectivity of the sensors means that they are able to react to the presence of many substances belonging to the same group of chemical compounds, but each of them has a different sensitivity towards a given substance present in the sample [26]. It means that the principle of operation of the sensor arrays is the overlapping of the activity ranges of individual sensors, and the output signal is multidimensional and unique for a given gas mixture [20]. Such an approach requires the use of multivariate data analysis methods that allow the determination of desired parameters of the sample on the basis of a multivariate set of signals from individual sensors of the matrix.

This article presents the process of development of a sensor array to monitor the dry methane reforming process. Reforming is one of the methods of obtaining syngas (a gaseous mixture of hydrogen and carbon monoxide). Syngas is one of the basic products of the chemical industry, and at the same time, a raw material for many important processes. Therefore, the development of technologies allowing to minimize the carbon footprint of traditional methods of energy generation is undoubtedly one of the greatest challenges of science.

One type of methane reforming is DRM (dry methane reforming). The process proceeds according to the following reaction:

(1) $$CH_{4}+CO_{2}⟶2CO+2H_{2}$$

An important approach is to obtain a carbon monoxide and hydrogen mixture with a molar ratio of 1:1 by this reforming. Dry methane reforming is a promising alternative to the currently used processes leading to the production of syngas with a given ratio of hydrogen to carbon monoxide. The main advantage of dry reforming is the use of two main greenhouse gases as raw materials—methane and carbon dioxide. The dry reforming process is carried out at atmospheric pressure, which is also a great advantage in terms of safety and simplicity of the construction of the reactors. The composition of the synthesis gas obtained is suitable for the Fischer–Tropsch synthesis and alternative, clean fuels for diesel engines.

The article presents the development of a gas sensor array for process monitoring of the dry methane reforming process, based on commercially available chemical sensors. The article describes the selection of sensors used in the matrix and validation of the matrix with the use of model gas mixtures that reflect the composition of the process streams of the reforming process, so it can be used on an industrial scale to supervise and control the ongoing process in real-time to automate process analytics. The use of sensor arrays for process control has a very high application potential. It allows for a significant reduction in the cost of process control while maintaining the assumed accuracy of the obtained results.

## 2. Materials and Methods

### 2.1. Chemical Gas Sensors

For the presented research, a set of commercially available chemical gas sensors was selected. Figaro Engineering Inc. sensors from the TGS series were used. Basic information on the sensors used is presented in Table 3.

### 2.2. Gas Mixtures Preparation

All gas mixtures were prepared in Tedlar gas sampling bags. Three-liter bags were used, and the total volume of the prepared mixtures was 2000 mL each time. In order to prepare gas mixtures, the following steps had to be taken:

(1) Assumption of the desired concentration of individual chemical substances in the gas mixture ($C_{m}$, ppm *v*/*v*),
(2) Assumption of the total volume of the gas mixture ($V_{m}$, mL),
(3) Determination of the volume of individual substances that must be dosed into the Tedlar bag ($V_{i}$, mL):
  (2) $$V_{i}=\frac{C_{m}\cdot V_{m}}{10^{6}},$$
(4) Determination of the air volume ($V_{0}$, mL), which must be dosed into the Tedlar bag:
  (3) $$V_{0}=V_{m}-\sum V_{i},$$

The volume of air dosed to the Tedlar bag was controlled with a RED-Y GSC-B9SS-B23 mass flow controller. Individual chemicals were taken from gas cylinders containing pure gases (>99.9%, Linde Gaz Polska) and then dosed to the Tedlar bag using Hamilton gas-tight syringes.

To determine the correctness of making the mixtures, all prepared gas mixtures were analyzed using a gas chromatograph (AutoSystem XL, Perkin-Elmer, Norwalk, CT, USA) equipped with a Porapak Q column (Sigma-Aldrich, Merck, Darmstadt, Germany), 100–120 mesh, OD 3.2 mm × 6.5 m, and a thermal conductivity detector (TCD). The oven temperature was set at 60 °C. Turbochrom software was used for recording and processing of chromatograms.

### 2.3. Gas Sensor Array Measurements

**Figure 1:**
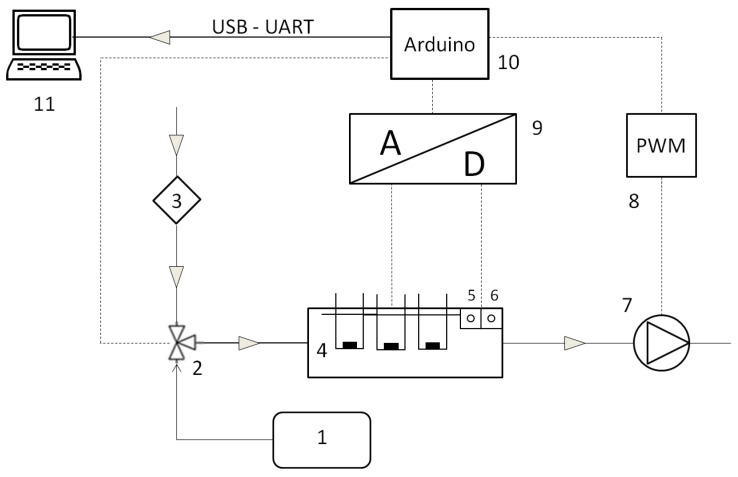
Gas sensor array testing system.

The diagram of the test equipment constructed for the sensor analysis is shown in Figure 1. It consisted of the following elements:(1) Tedlar foil bag, (2) three-way valve, (3) air filter, (4) gas sensor array, (5) temperature sensor, (6) humidity sensor, (7) diaphragm pump, (8) pulse width modulation module, (9) analog-to-digital converter, (10) Arduino MEGA2560 platform, (11) computer. A stream of clean air flowed through the measuring chamber at a constant flow rate, controlled by the diaphragm pump rotation speed. The change in the pump rotation is caused by a change in the DC voltage supplied to the pump motor. The PWM module (8) controlled by Arduino (10) is responsible for the regulation of the supply voltage. By changing the position of a three-way valve (2), a sample of the prepared gas mixture was directed to the measuring chamber. After completion of the measurement, clean air was returned to the measuring chamber in order to regenerate the sensors by changing the valve (2) position. Flushing the sensors with clean air made it possible to restore them to their initial parameters and prepare them for subsequent analyzes. The electrical systems for each of the sensors have been prepared in accordance with the manufacturer’s requirements. The signals from the sensors were recorded using an analog-to-digital converter (ADS1015). All data were saved on the computer using dedicated software.

**Figure 2:**
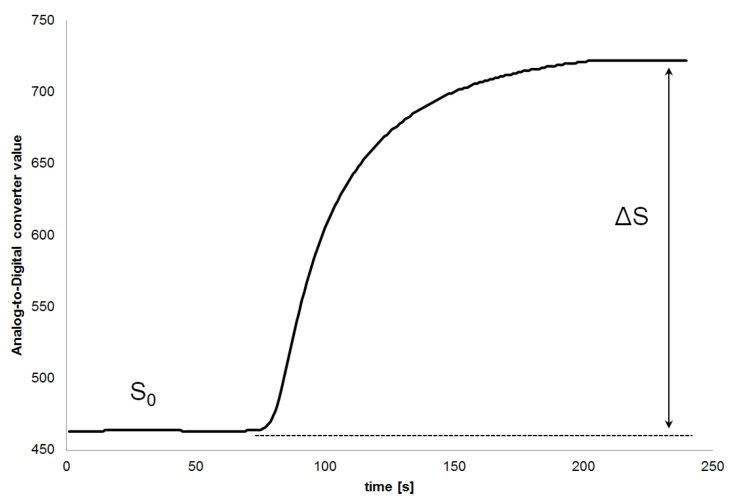
Example of the sensor response.

An example of the sensor response is shown in Figure 2. For data analysis, the relative sensor response ($S_{R}$) was used. It was calculated using the formula:

(4) $$S_{R}=\frac{\Delta S}{S_{0}}$$

### 2.4. Data Analysis

Data analysis and other calculations were performed using RStudio Desktop (v. 1.0.143) software. Principal Component Regression (PCR) was selected as the calibration model for the gas sensor array. The PCR method tutorial in R language is presented on the website [27]. The chemical gas sensors are usually responsive to multiple gases, which would make them individually quite useless. For this reason, the PCR method was chosen as the data analysis method. It enables the deconvolution of the collected sensor data sets and minimizes the impact of low selectivity of sensors in the matrix. It should be noted that other methods of data analysis, such as Multiple Linear Regression (MLR) [28], Partial Least Squares Regression (PLSR) [29], or Artificial Neural Network (ANN) [30], can also be successfully used for this type of application.

This model assumes reducing the number of explanatory variables by selecting few first principal components (PCs) in the place of the primary variables. The guiding idea of the PCR method is to formulate a relationship between PCs and the expected concentration of the component. The method consists of two steps:

(1) Determination of the principal components using the principal component analysis (PCA) method. It allows obtaining an uncorrelated matrix of variables.
(2) Development of the Multiple Linear Regression (MLR) model with the use of principal components as variables.

**Figure 3:**
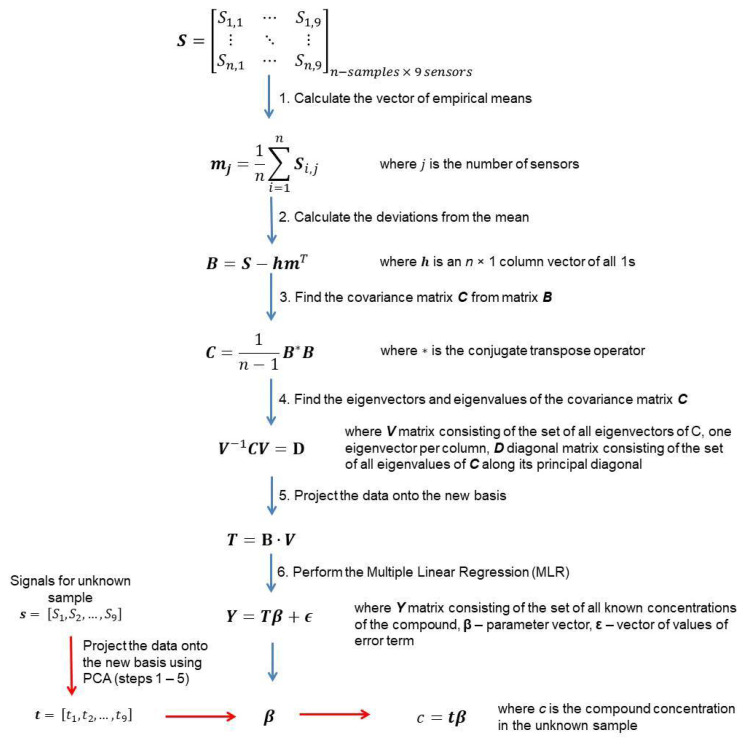
The PCR algorithm.

A detailed representation of the PCR algorithm is presented in Figure 3.

## 3. Results

**Figure 4:**
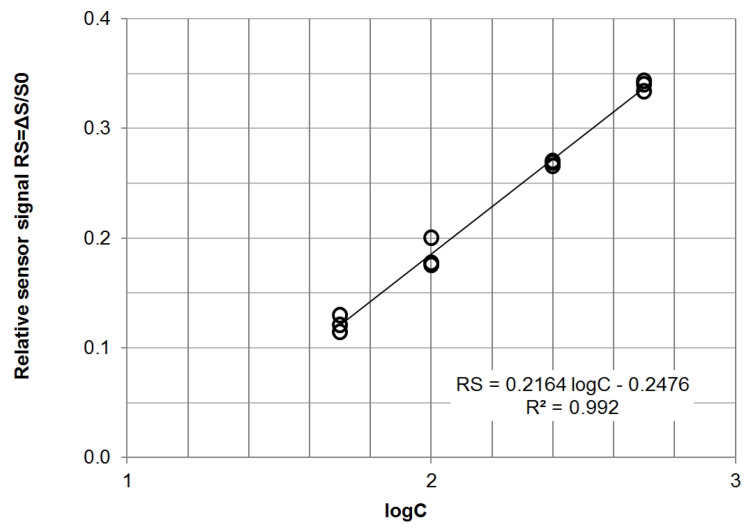
Static characteristics of the Figaro TGS2600 sensor in the presence of carbon monoxide.

In the first stage of the research, the static characteristics of all tested sensors were collected. For this purpose, mixtures of single compounds (methane, carbon dioxide, carbon monoxide and hydrogen) with air in the concentration range of 50–1000 ppm *v*/*v* were prepared. An example of the response characteristics to the presence of carbon monoxide determined for the TGS2600 sensor is shown in Figure 4. In order to ensure the linearity of the characteristics, they were plotted in the S=f(logC) coordinates, where *S* is the signal obtained from the analog-to-digital converter, and *C* is the gas concentration in the mixture with air (ppm *v*/*v*).

Based on the collected static characteristics, the sensitivity of each sensor to a single gas was determined. The values obtained are presented in Table 4.

Based on the obtained sensitivity values, a set of sensors for the matrix was selected. Only the catalytic sensors (TGS6810 and TGS6812) were rejected from the tested set due to the low sensitivity values in the tested concentration range. These sensors are used in the presence of high concentrations of explosive gases. Note that it is necessary to use electrochemical sensors TGS4161 and TGS5042, which show high sensitivity and selectivity towards carbon dioxide and carbon monoxide, respectively. The sensitivity values obtained for the remaining sensors indicate the correct selection of sensor models. All of them react to the presence of the tested gases.

In the next step, model gas mixtures were prepared to reflect the expected inlet and outlet concentrations from the dry methane reforming process. The sensor matrix responses to all possible concentration combinations shown in Table 5 were recorded, repeating each analysis three times. The DRM inlet stream is usually a pure mixture of methane and carbon dioxide; therefore, binary mixtures were used as inlet mixtures. In the case of outlet streams, the possible incomplete conversion of all substrates must be taken into account. Therefore, the outlet mixtures were prepared as four-component mixtures.

**Figure 5:**
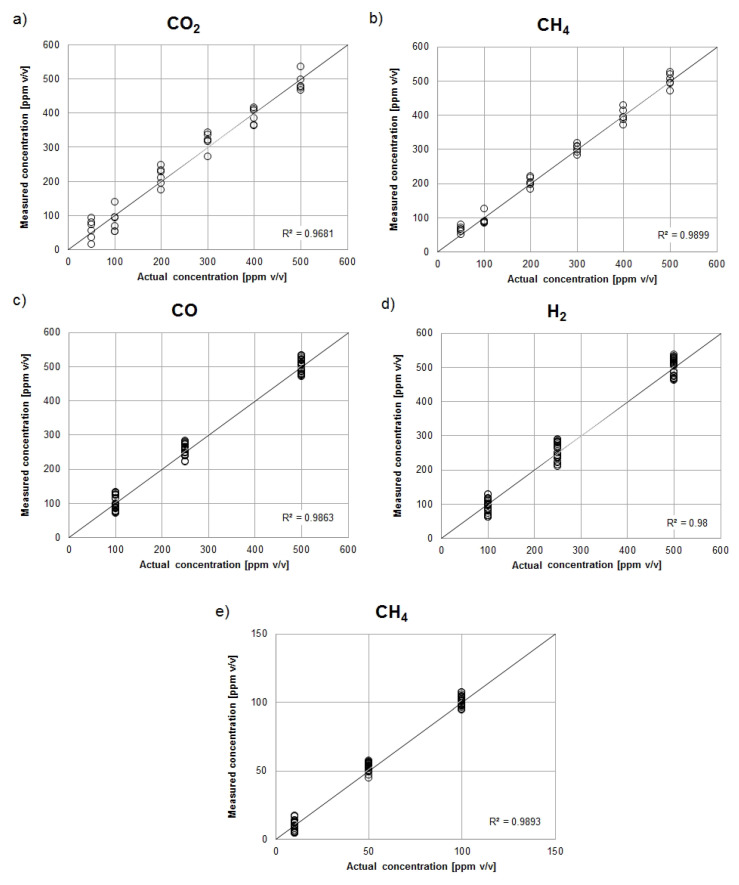
Validation plots of PCR calibration models for DRM inlet stream: (**a**) carbon dioxide, (**b**) methane and DRM outlet stream: (**c**) carbon monoxide, (**d**) hydrogen, (**e**) methane.

Based on the recorded matrix response signals, five PCR calibration models were developed: for the inlet stream (carbon dioxide and methane models) and for the outlet stream (carbon monoxide, hydrogen, and methane models). The validation of these models was performed on the basis of correlation charts presented in Figure 5.

In the last stage of the research, gas mixtures simulating the actual compositions of process mixtures were prepared. The concentrations of individual components were determined using developed PCR models. The determined concentrations were converted into the following parameters characterizing the course of the dry methane reforming process:

1. Inlet molar ratio (IMR):
  (5) $$IMR=\frac{C_{CO_{2}}}{C_{CH_{4}}}$$
2. Outlet molar ratio (OMR):
  (6) $$OMR=\frac{C_{H_{2}}}{C_{CO}}$$
3. Methane conversion level (MCL):
  (7) $$MCL=\frac{C_{CH_{4},in}-C_{CH_{4},out}}{C_{CH_{4},in}}\cdot 100\%$$

**Figure 6:**
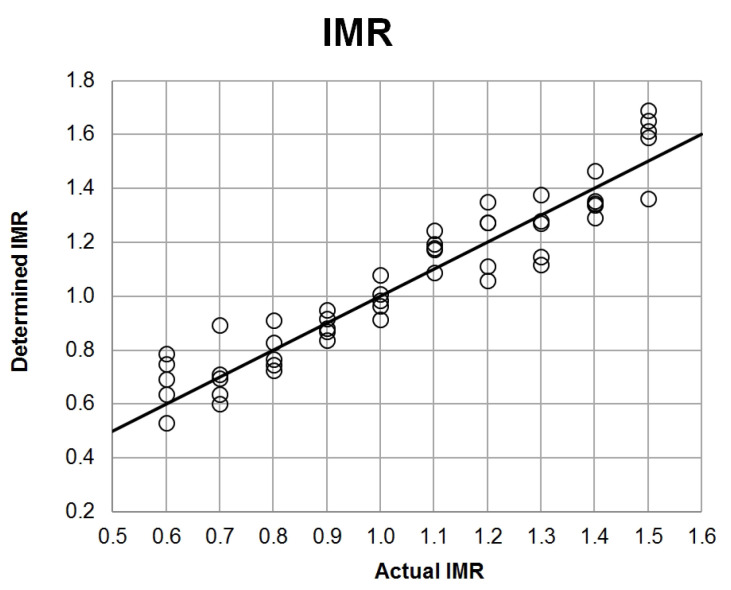
Actual and determined Inlet Molar Ratio (IMR) correlation plot.

Figure 6 presents the IMR parameter determination using a gas sensor array in the range from 0.6 to 1.5. The Root-Mean-Square Error (RMSE) for the entire range is equal to 0.096. However, in the range 0.9–1.1, where these IMR values are most commonly found in industrial conditions, the RMSE is equal to 0.065.

**Figure 7:**
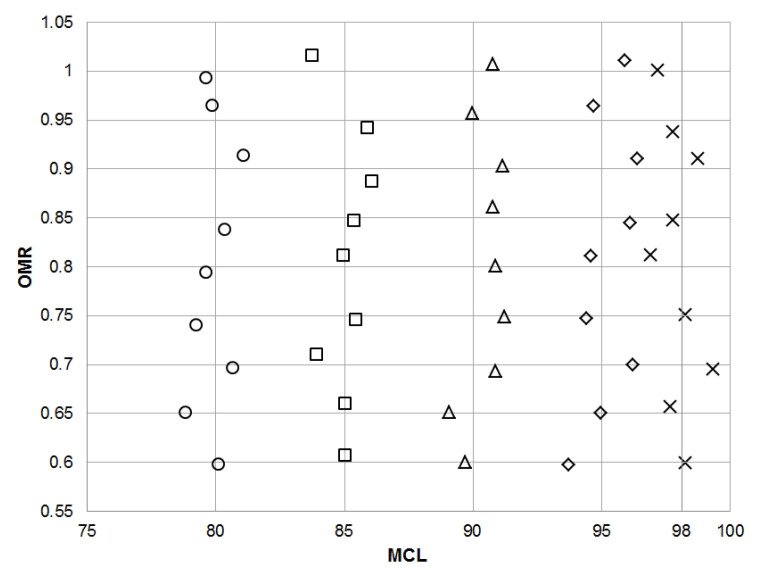
Actual and determined Inlet Molar Ratio (IMR) correlation plot.

Figure 7 presents the OMR and MCL values determined using a gas sensor array. They were measured using model gas mixtures, the composition of which was represented by the values of parameters equal to the intersections of the gridlines in Figure 7. Model gas mixtures with composition within the range of parameters: OMR 0.6–1.0 (step 0.5) and MCL 80–95% (step 5%) and 98% were used. The RMSE for MCL determination is equal to 0.8%, and for OMR determination, it is equal to 0.008.

## 4. Discussion

The article presents a new method of monitoring and assessing the course of the dry methane reforming process with the use of a gas sensor array. Nine commercially available gaseous chemical sensors were used to construct the array. Such a solution enables the repeated production of the developed array, as opposed to solutions based on prototype sensors.

The performed tests of the sensitivity of sensors for the process mixtures components allowed to reject sensors that showed the lowest sensitivity in the assumed concentration range. These were the catalytic sensors TGS6810 and TGS6812. Two types of sensors were used in the final solution: electrochemical (2 pieces) and metal oxide sensors (7 pieces).

The array constructed in this way was calibrated to determine the concentrations of methane, carbon dioxide, carbon monoxide and hydrogen. Model gas mixtures representing the actual streams of the methane reforming process were used for calibration. Principal Component Regression (PCR) was used as a calibration method. The validity of the use of this method is confirmed by the values of the determination coefficients ($R^{2}$) of the correlation plots (Figure 5), the value of which in all five cases was higher than 0.95.

The developed PCR models were used to determine the quantitative parameters of the description of the reforming process: Inlet Molar Ratio (IMR), Outlet Molar Ratio (OMR), and Methane Conversion Level (MCL). In the case of the input parameter (IMR), the mean error in determining this parameter is 0.096 for the range of molar ratios 0.6–1.5. However, in the case of the process range (0.9–1.1), this error is 0.065, which is about 6.5% of the measured value. The OMR and MCL values were determined for the mixtures representing outlet streams of the reforming process. For the molar ratio, an average error of 0.008 was obtained (which gives about 0.8% of the measured value), while for the conversion degree, the average error was 0.8%.

Such results are very promising. They show that the use of an array of non-selective chemical sensors together with an appropriately selected mathematical model can be used in the monitoring of commonly used industrial processes, such as, for example, methane reforming. However, it should be noted that these matrices cannot be used directly in the process stream. In the case of the presented research, all analyzes were carried out on model gas mixtures with component concentrations of up to about 1000 ppm *v*/*v* in air, under atmospheric pressure, at room temperature. In the actual reforming process, the streams have a temperature of 650–850 °C (in the case of dry reforming), and their composition is expressed in tens of percent. For this reason, the next step in future research should be to check the possibility of using the array in real conditions, using a sample cooler and a dilution module that would reduce gas concentrations to the required ranges and also enable the operation of metal oxide semiconductor sensors, which require oxygen for proper operation. However, the cooling and dilution processes are basic operations that are performed in the case of process analytics; therefore, it can be assumed that the constructed array in this way can also be successfully used in real industrial conditions. An additional advantage is the low price of the sensors, compared to expensive chromatographic analyzers that require periodic maintenance and ensure the availability of high purity gases. According to the sensor manufacturer’s declaration, their lifetime is about 2 years, which ensures a relatively long time of maintenance-free use.

The proposed solution can also be used to monitor biogas-fed processes. In such an application, the proposed matrix should be supplemented with sensors detecting hydrogen sulfide, ammonia, or nitrogen oxides. In this case, it is best to choose electrochemical sensors (for example, the Figaro FECS series) that allow the determination of these components at low concentration levels. The use of additional sensors requires additional calibrations using the PCR method, but thanks to this, it will also be possible to simultaneously monitor two processes—biogas purification and methane reforming.

In terms of the accuracy of the obtained results, the sensor matrices do not match the chromatographic methods, but in cases where the accuracy of the obtained results does not have to be high, they are a very good and, at the same time, cheap alternative to specialized instrumental techniques. The comparison of the constructed sensor matrix with commercially available solutions is presented in Table 6.

## 5. Conclusions

The article presents a new method of monitoring the dry methane reforming process with the use of a matrix of chemical sensors. The matrix consisted of nine commercially available sensors (7 metal oxide sensors and 2 electrochemical) produced by Figaro Engineering Inc. Principal Component Regression (PCR) was used as a calibration method. The tests were performed on 117 model gas mixtures, characterized by the following process parameters: Inlet Molar Ratio (IMR) in the range 0.6–1.5, Outlet Molar Ratio (OMR) in the range 0.6–1.0, and Methane Conversion Level (MCL) in the range 80–95%. The best results (compared to gas chromatography) were obtained for OMR and MCL parameters—they did not exceed 1% of the parameter value. In the case of the IMR parameter, this error was about 7%.

We conclude that sensor matrices can be used on an industrial scale to supervise and control the ongoing process in real-time in order to automate process analytics. The use of sensor and sensor arrays for process control is becoming more and more popular and has a very high application potential, i.e., monitoring of biofiltration processes [28], bioreactors [39,40,41], quality assurance in the pharmaceutical industry [42], food industry [43,44] and odour monitoring [45]. It allows for a significant reduction in the cost of process control while maintaining the assumed accuracy of the obtained results.

## Figures and Tables

**Table 1:** Examples of the use of gas sensor arrays to monitor industrial processes.

Sensor Array	Number of Sensors	Process	Reference
An infrared matrix sensor	1	Monitor the FC stack temperature distribution	[3]
An array of thin film tin oxide sensors prepared by RF sputtering onto alumina and doped with chromium and indium	16	Wine classification and prediction based on an electronic nose (e-nose)	[4]
Metal oxide semiconductors (MOSs) and Metal Oxide semiconductor field-effect transistors (MOSFETs)	50	Monitors bioreactors and highlights their potential for controlling quality and safety, and for the optimization and automatic control of bioprocesses	[5]
MOSFET-sensors with catalytic metal gates of palladium, iridium or platinum	10	Non-invasive monitoring of the physiological changes in fermentation processes	[6]
Five TGS sensors from Figaro, Japan (TGS-832, TGS-823, TGS-2600, TGS-2610 and TGS-2611)	5	Predicting the optimum fermentation time at an earlier stage of the process	[7]
Sensor array of different types of metal oxide gas sensor (MOSs)	8	Study the tempeh fermentation process and the stages of this process	[8]
Sensor array was comprised of five sensors supplied by Figaro (Japan) and five sensors obtained from HANWIE Electronics (China)	10	Identification of different types of saffron, stigma of Crocus sativus, based on their volatile organic compounds (VOCs)	[9]
Semiconducting tin dioxide based sensors and an optical carbon detector	4	Monitoring an ethanol batch cultivation with the yeast *Saccharomyces cerevisiae*	[10]
Metal oxide sensor arrays	10	Prediction of the alcohol content of the green jujube wine fermentation	[11]
Sensor array containing different gas-sensitive semiconductor devices and an infrared gas sensor	14	Measuring the emission from a production-scale baker’s yeast manufacturing process and monitor the gas emission from a yeast culture bioreactor during fed-batch operation	[12]
Metal oxide sensor arrays	9	Determine the fermentation degree of cocoa beans	[13]
Metal oxide semiconductors (MOS) chemical sensors	18	Identification of different fermentation times and bile species of Bile Arisaema	[14]
Potentiometric sensor array: polymeric cation-sesnitive (8), polymeric anion-sensitive (8) and metallic and chalcogenide glass sensor with RedOx sensitivity	23	Real-time monitoring of ammonium and nitrate nitrogen in processed water at aeration plant	[15]
Hybrid sensor array composed by InterDigitated Chemocapacitora (IDVc) with the appropriate read-out electronic	8	The monitoring and evaluation and control of the specific Volatile Organic Compounds (VOCs)	[16]

**Table 2:** Advantages and disadvantages of gas chromatography and sensor arrays.

Analysis Method	Advantages	Disadvantages
Gas chromatography	Enables qualitative and quantitative analysis at the same time High repeatability, reproducibility and accuracy Possibility to identify all compounds present in the mixture High resolution	Limited measuring ranges and resulting from them a limit of quantification of individual compounds Impossible to predict interactions between components The necessity to store and transport of the samples The representativeness and integrity of a sample depends on many factors High costs
Gas sensor arrays	Enables continuous process monitoring Sensor array in the form of easily replaceable modules Uniqueness of the generated signal (a different signal for each tested mixture) Low costs Easy sensors calibration	No qualitative analysis possible High influence of temperature and humidity on measurement stability Multidimensionality of the generated signal (requires averaging using statistical methods) Complicates signal identification system (graphic methods, analytical procedures, neural network)

**Table 3:** Basic characteristics of Figaro Engineering Inc. gas sesnors.

Sensor Type	Model	Detected Gases
Catalytic	TGS6810	methane, propane, iso-butane
Catalytic	TGS6812	methane, propane, hydrogen
Electrochemical	TGS4161	carbon dioxide
Electrochemical	TGS5042	carbon monoxide
Metal Oxide Semiconductor	TGS2600	methane, carbon monoxide, hydrogen
Metal Oxide Semiconductor	TGS2602	hydrogen, toluene, ethanol
Metal Oxide Semiconductor	TGS2603	hydrogen, ethanol
Metal Oxide Semiconductor	TGS2611	ethanol, hydrogen, methane
Metal Oxide Semiconductor	TGS3870	carbon monoxide, methane
Metal Oxide Semiconductor	TGS823	carbon monoxide, methane, iso-butane
Metal Oxide Semiconductor	TGS8100	methane, hydrogen, ethanol

**Table 4:** Sensitivity of the tested sensors.

	${\mathrm{CH}}_{4}$	${\mathrm{CO}}_{2}$	CO	$H_{2}$
TGS2600	0.114	0.034	0.216	0.332
TGS2602	0.089	0.022	0.091	0.087
TGS2603	0.082	0.025	0.085	0.221
TGS2611	0.196	0.015	0.102	0.146
TGS3870	0.165	0.012	0.033	0.074
TGS4161	0.012	0.307	0.013	0.022
TGS5042	0.019	0.009	0.247	0.042
TGS6810	0.005	0.002	0.005	0.005
TGS6812	0.004	0.001	0.006	0.005
TGS823	0.211	0.019	0.233	0.185
TGS8100	0.052	0.011	0.138	0.201

**Table 5:** Compositions of tested gas mixtures.

	DRM Inlet Stream	DRM Outlet Stream
total number of mixtures	36	81
$CH_{4}$	50, 100, 200, 300, 400, 500 ppm *v*/*v*	10, 50, 100 ppm *v*/*v*
$CO_{2}$	50, 100, 200, 300, 400, 500 ppm *v*/*v*	10, 50, 100 ppm *v*/*v*
CO	-	100, 250, 500 ppm *v*/*v*
$H_{2}$	-	100, 250, 500 ppm *v*/*v*

**Table 6:** The comparison of the constructed sensor matrix with commercially available devices.

Producer	Model	Technology	Range	Accuracy	Response Time	Reference
Gdańsk University of Technology	Sensor matrix prototype	MOS and EC gas sensors	CO, $CO_{2}$, $CH_{4}$, $H_{2}$: 0–100% (using dilution system)	5% Full Scale (FS)	<90 s to 90% step range (MOS)	-
Cubic Sensor and Instrument Co.	Portable Infrared Syngas Analyzer Gasboard-3100P	CO, $CO_{2}$, $CH_{4}$ (NDIR) $H_{2}$ (TCD)	CO: 0–30% $CO_{2}$: 0–25% $CH_{4}$: 0–10% $H_{2}$: 0–30%	2% Full Scale (FS)	<15 s to 90% step range (NDIR)	[31]
Cubic Sensor and Instrument Co.	Syngas Analysis System Gasboard-9021	CO, $CO_{2}$, $CH_{4}$ (NDIR) $H_{2}$ (TCD)	CO: 0–30% $CO_{2}$: 0–25% $CH_{4}$: 0–10% $H_{2}$: 0–30%	CO, $CO_{2}$, $CH_{4}$ < 1% FS $H_{2}$ <2% FS	<15 s to 90% step range (NDIR)	[32]
Hubei Cubic-Ruiyi Instrument CO.	Portable Natural Gas Analyzer Gasboard-3110P	$CO_{2}$, $CH_{4}$ (NDIR)	$CO_{2}$: 0–5% $CH_{4}$: 0–100%	<2% FS	<15 s to 90% step range (NDIR)	[33]
Nova Analytical Systems (a Unit of Tenova Goodfellow Inc.)	970P Portable Multi-Gas Industrial Analyzers	CO, $CO_{2}$, $CH_{4}$ (NDIR) $H_{2}$ (TCD)	CO: 0–2% or 0–50% $CO_{2}$: 0–2% or 0–50% $CH_{4}$: 0–2% or 0–50% $H_{2}$: 0–2% or 0–50%	0.1% for all gases <1% FS in 8 h	20–30 s to 90% step range	[34]
VASTHI Instruments Pvt Ltd	Online Syngas Analyzer by Vasthi	CO, $CO_{2}$, $CH_{4}$ (NDIR) $H_{2}$ (TCD)	CO: 0–100% $CO_{2}$: 0–100% $CH_{4}$: 0–50% $H_{2}$: 0–50%	CO, $CO_{2}$, $CH_{4}$: 0.5% from range or ± 3% $H_{2}$: ±5 ppm or 5%	45 s to 90% step range	[35]
Wuhan Tianyu Intelligent Control Technology Co., Ltd. (TIANYU)	Syngas Analyzer Portable SYN-600	CO, $CO_{2}$, $CH_{4}$ (NDIR) $H_{2}$ (TCD-MEMS)	CO: 0–100% $CO_{2}$: 0–100% $CH_{4}$: 0–100% $H_{2}$: 0–100%	CO, $CO_{2}$, $CH_{4}$: ±2% FS $H_{2}$: ±3% FS	45 s to 90% step range	[36]
MRU GmbH	SWG 100 Syngas	CO, $CO_{2}$, $CH_{4}$ (NDIR) $H_{2}$ (TCD)	CO: 0–10/100% $CO_{2}$: 0–10/100% $CH_{4}$: 0–10/100% $H_{2}$: 0–10/100%	no data	no data	[37]
ETG Risorse e Technologia S.r.l.	MCA 100 SYN P – Portable Syngas Analyzer	CO, $CO_{2}$, $CH_{4}$ (NDIR) $H_{2}$ (TCD)	Modified according to the needs of customer	CO, $CO_{2}$, $CH_{4}$, $H_{2}$: ±2% FS	no data	[38]
